# Understanding the Human Toll of Human-Elephant Conflicts: Insights From a Four-Year Autopsy Analysis

**DOI:** 10.7759/cureus.76763

**Published:** 2025-01-01

**Authors:** Ajay Bhengra

**Affiliations:** 1 Forensic Medicine, Sheikh Bhikhari Medical College, Hazaribagh, IND

**Keywords:** corridors, fatalities, forest conservation, human-elephant conflict, wildlife

## Abstract

Introduction

Not only does attention to the delicate natural balance of elephant and human coexistence spawn progress, but it also rightly draws attention. In that case, it can benefit both pockets of biodiversity and facilitate healthier ecosystems and a more sustainable future for elephants and people alike. Yet those human-elephant conflicts (HECs) have increased as habitat degradation and urbanization have repeatedly cut elephant pathways. These conflicts harm local communities and locally assisted conservation because of many fatalities, crop destruction, and property damage. To mitigate these accidents and encourage cohabitation, trend information and contributing factors to HEC deaths are needed. We can make our future an elephant and a person's shared life if we have the correct information and do the right thing.

Materials and methods

This cross-sectional note analyzes an autopsy-derived study of HEC victims from the tertiary care center, Hazaribagh, Jharkhand, between January 2020 and December 2023. The decomposed bodies, along with deaths from other causes, were excluded, and fatalities caused by HEC were the inclusion criteria. Victims were aged, sexed, and placed based on the month and year they died. The frequency and distribution of these variables were analyzed using IBM SPSS Version 27 (IBM Corp, Armonk, NY). Finally, data were classified and summarized to reveal events that consistently coincided with HEC-related deaths.

Results

In the four-year study period, there were 23 HEC-related deaths reported. The victim was male in 52.2% and female in 47.8%. The most affected age groups were 41-50, 31-40 (17.4%), and 51-60 (17.4%), respectively. In 2021 (47.8%) and 2022 (21.7%), there were the highest fatalities, followed by 2023 (17.4%) and 2020 (13.0%). Of the five identified, October had the highest cases (21.7%), with February, April, and December making 17.4% each. The incidence was lowest in May and August (4.3% each) and second lowest in July and September (8.7% each). Deaths tended to occur between 4 PM and 12 AM (47.8%); the next most prominent peak was 12 AM to 8 AM (34.8%). Most died (91.3%) at the scene of the incident, with two patients (8.7%) dying at medical facilities. Death was primarily due to combined head injury and hemorrhagic shock (91.3%).

There was a significant association between year and month of incidence (χ²(21) = 47.44, p = 0.001), suggesting nonrandom patterns in fatalities. Deaths of patients accounted for the highest monthly fatality rate (45.5%; October 2021).

Conclusion

Results show the need to mitigate HEC in Jharkhand. The strong association between fatalities and the year and month of fatalities shows seasonal and temporal patterns with these conflicts. Improved public awareness, enhanced management of elephant corridors, and planning for urbanization are indispensable to avoid HEC incidents. While this sounds great, the critical role of stakeholder collaboration makes everyone's involvement imperative for effectively overcoming these conflicts for the coexistence of humans and elephants.

## Introduction

Elephants and humans have lived together on the earth for centuries in harmony with nature [1]. The Asian elephant, *Elephas maximus*, is legally protected and awe-inspiring. The Indian Wildlife Protection Act (1972) provides Schedule 1 and Part 1 as protection for wildlife; above all, this holds the highest level of security. Throughout many cultures, elephants have been revered and honored. They are sacred to other communities and have considerable cultural value for them. Article 48A of Part IV of the Indian Constitution compels the State to protect its forests and wildlife. Article 51A of Part IVA also holds an Indian citizen to a fundamental duty to contribute to this conservation [2]. This cultural significance of elephants has cemented our resolve to fight for those we love, in this case, elephants.

There are many causes of wildlife-human conflicts, including the illegal wildlife trade, driven by the demand for ivory, poaching for financial gains, clearing forests for agriculture, logging, or development that reduce elephants' natural habitats. The impact of human settlements, road and railway construction, and mining activity drives elephants in forested regions to become aggressive. A big part of the cause of these conflicts, though, is disrupting their natural migratory paths. Elephant corridors are narrow paths connecting big ones needed for movement. Most of it is due to urban expansion, agricultural development, and unregulated project growth (e.g., shopping malls or housing estates). In 2005, 45.5% of corridors were less than 1 km wide; in 2017, this increased to more than 74% [3].

Humans have conflicts with elephants (human-elephant conflicts, HECs) because of crop destruction, property damage, and death. These conflicts contribute to local animosity and, hence, harm conservation efforts. They also lead to the retaliatory killing of elephants. For this reason, it is important to understand HEC in many contexts in which swift response to growing conflicts is demanded [4].

"However, the World Wildlife Fund reports that in India, elephants are to blame for more than 100 human deaths a year," reported CNN [5]. Federal and State governments spend significant resources to manage devastating incidents and to compensate people affected ex gratia [6].

One primary goal of the study was to measure the incidence region. This initiative aims to raise public and government bodies' awareness of such events and increase knowledge on future human-wildlife conflict prevention. Understanding these conflicts can help us solve them.

## Materials and methods

Study site

This retrospective cross-sectional analysis occurred over four years at the Department of Forensic Medicine and Toxicology.

Ethical statement

The Institutional Ethics Committee, Sheikh Bhikhari Medical College approved the study vide letter no SBMC/IEC/2024/12 dated 23/04/2024. The committee emphasized complying with the ethical guidelines and standards including the protection of privacy and confidentiality of the victims.

Study size

The study was conducted at the Department of Forensic Medicine and Toxicology from January 2020 to December 2023. The only facility for autopsies on any person who has been brought dead to the district is this tertiary healthcare center in Hazaribagh district. The relatives of the victims and the accompanying police were interviewed, and detailed information regarding the HEC was taken. The details regarding the place and time of the incident, the circumstances of the elephant confrontation, attempts to save the victims, any measures taken by the local or forest authorities after the incident to chase away elephants into the forest, compensation, etc., were collected case-wise and incorporated together.

Inclusion/exclusion criteria

Fatalities brought to the mortuary for autopsy were part of the data collection. The study did not include severely decomposed bodies or whose cause of death was unrelated to HEC.

Data analysis

Documented details on the victims and case reports give details of the victim’s age and gender, the place of death, the cause of death, and the time, month, and year of HEC. After importing data from Excel spreadsheets, descriptive statistical analysis was studied using IBM SPSS version 27.0 (IBM Corp, Armonk, NY). All of the variables' frequencies were analyzed. Deaths by year and month of occurrence were analyzed using crosstabulation. The year and month of occurrence were subjected to chi-square tests. The findings were then compared to those from previous similar studies.

## Results

The gender distribution of fatalities

The four-year study reported 23 HEC fatalities during its duration. Among the 23 victims, 12 (52.2%) were male and 11 (47.8%) female (Table 1).

**Table 1:** Gender distribution of victims (N = 23)

Gender	Frequency	Percent
Male	12	52.2
Female	11	47.8
Total	23	100

The age-group-wise distribution of fatalities

**Figure 1:**
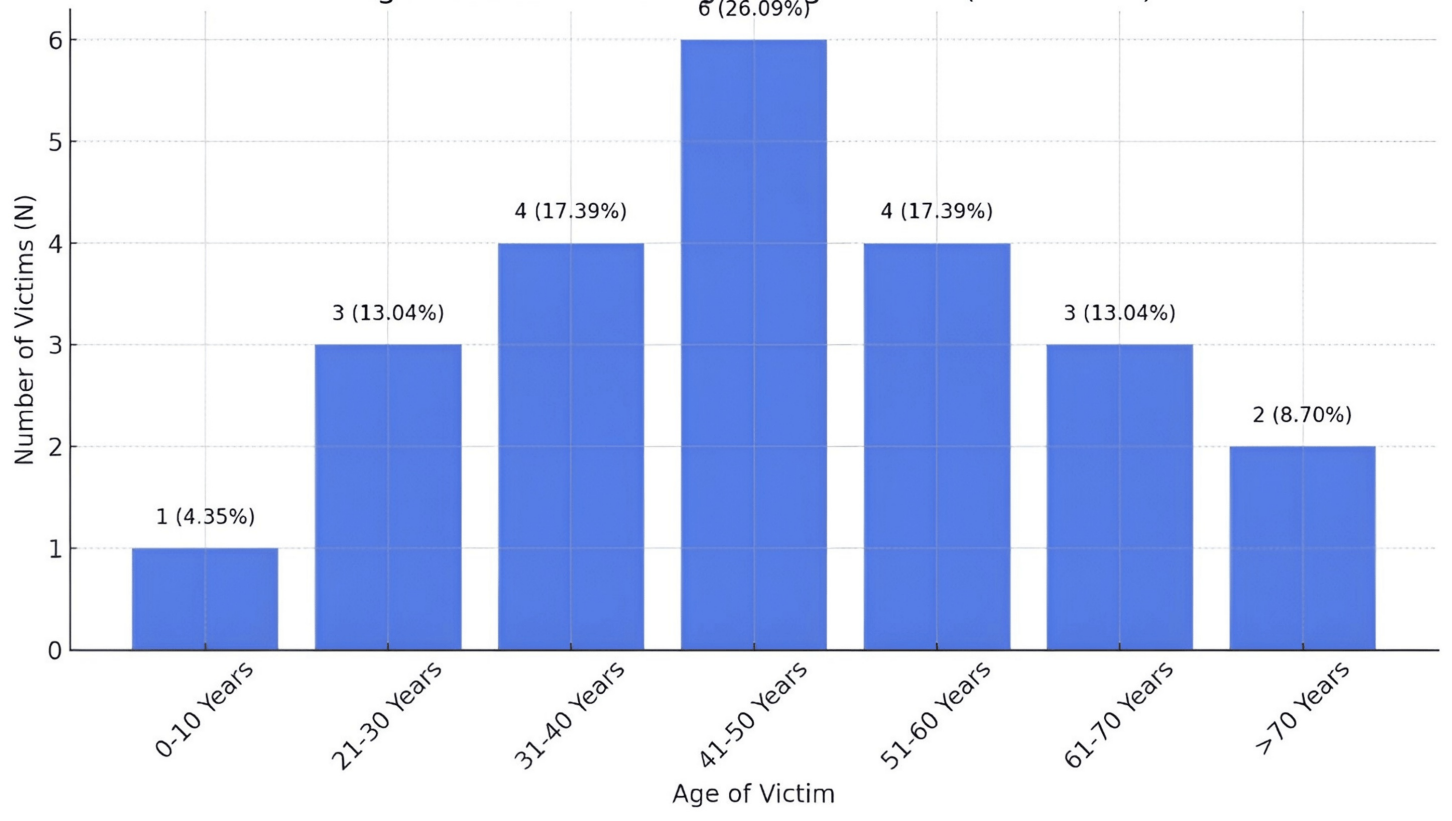
Age-group-wise distribution of HEC fatalities (N = 23) HEC, human-elephant conflict.

What was reassuring was that the number of victims under 21 years was small, with one (4.3%) falling in the 0-10 years age group. Victims' age included six (26.1%) of all victims, with age 41-50 years being the most common age group. Notable proportions were seen in other age groups, e.g. 31-40 years, four (17.4%), and 51-60 years, four (17.4%). Individuals aged 21-30 years had smaller percentages, three (13.0%); 61-70 years, three (13.0%); and 71 plus 2 (8.7%) (Figure 1).

The year-wise distribution of fatalities

**Figure 2:**
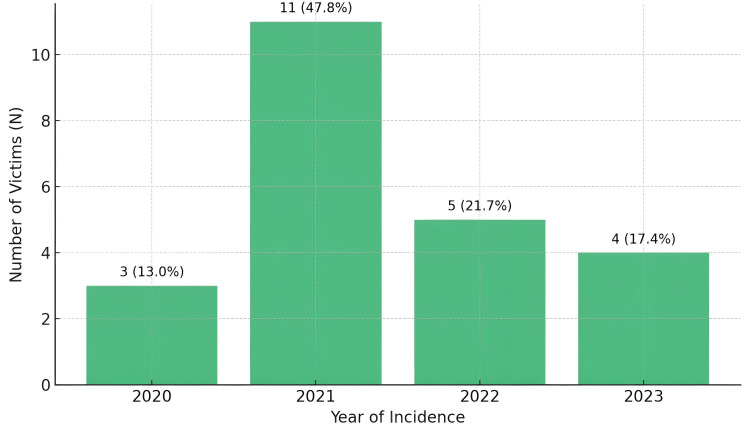
Yearly distribution of HEC fatalities (N = 23) HEC, human-elephant conflict.

In 2021, the proportion of fatalities was the highest, 11 (47.8%), of all cases. For example, five (21.7 %) deaths in 2022, four (17.4%) deaths in 2023, and three (13.0%) deaths in 2020 (Figure 2).

The month-wise distribution of fatalities

**Figure 3:**
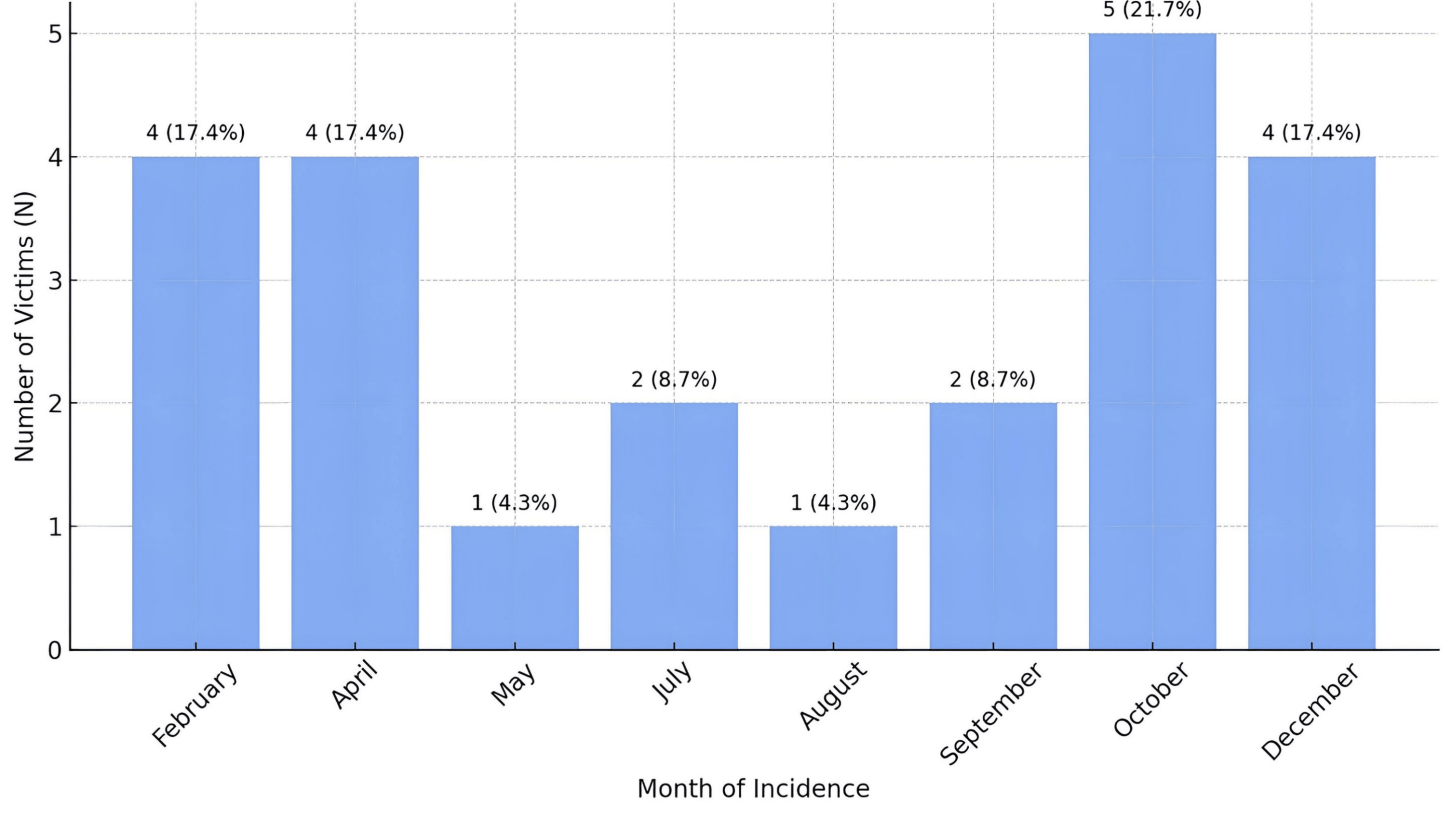
Monthly distribution of HEC fatalities (N = 23) HEC, human-elephant conflict.

The casualties were highest in October, with five (21.7%) cases. There were four (17.4%) fatalities each in February, April, and December. In July and September, the cases were two (8.7%) each; May and August were the least fatal, with one case (4.3%) (Figure 3).

The distribution of fatalities according to time of incidence

The incidence was most significant from 4 PM to 12 AM, with 11 (47.8%) fatalities. Between 12 AM and 8 AM, eight (34.8%) incidents were fatal, while four (17.4%) occurred between 8 AM and 4 PM (Table 2).

**Table 2:** Incidence timewise distribution of HEC fatalities HEC, human-elephant conflict.

Time of incidence	Frequency (N)	Percentage
12 AM-8 AM	8	34.8
8 AM-4 PM	4	17.4
4 PM-12 AM	11	47.8
Total	23	100

The distribution of fatalities according to place of death

Of the deaths, 21 (91.3%) occurred at the incident scene. Two (8.7%) died at a casualty or trauma center (Table 3).

**Table 3:** Place of death of victim

Place of death	Frequency	Percent
Spot	21	91.3
Casualty/Trauma center	2	8.7
Total	23	100

The distribution of fatalities according to cause of death

**Figure 4:**
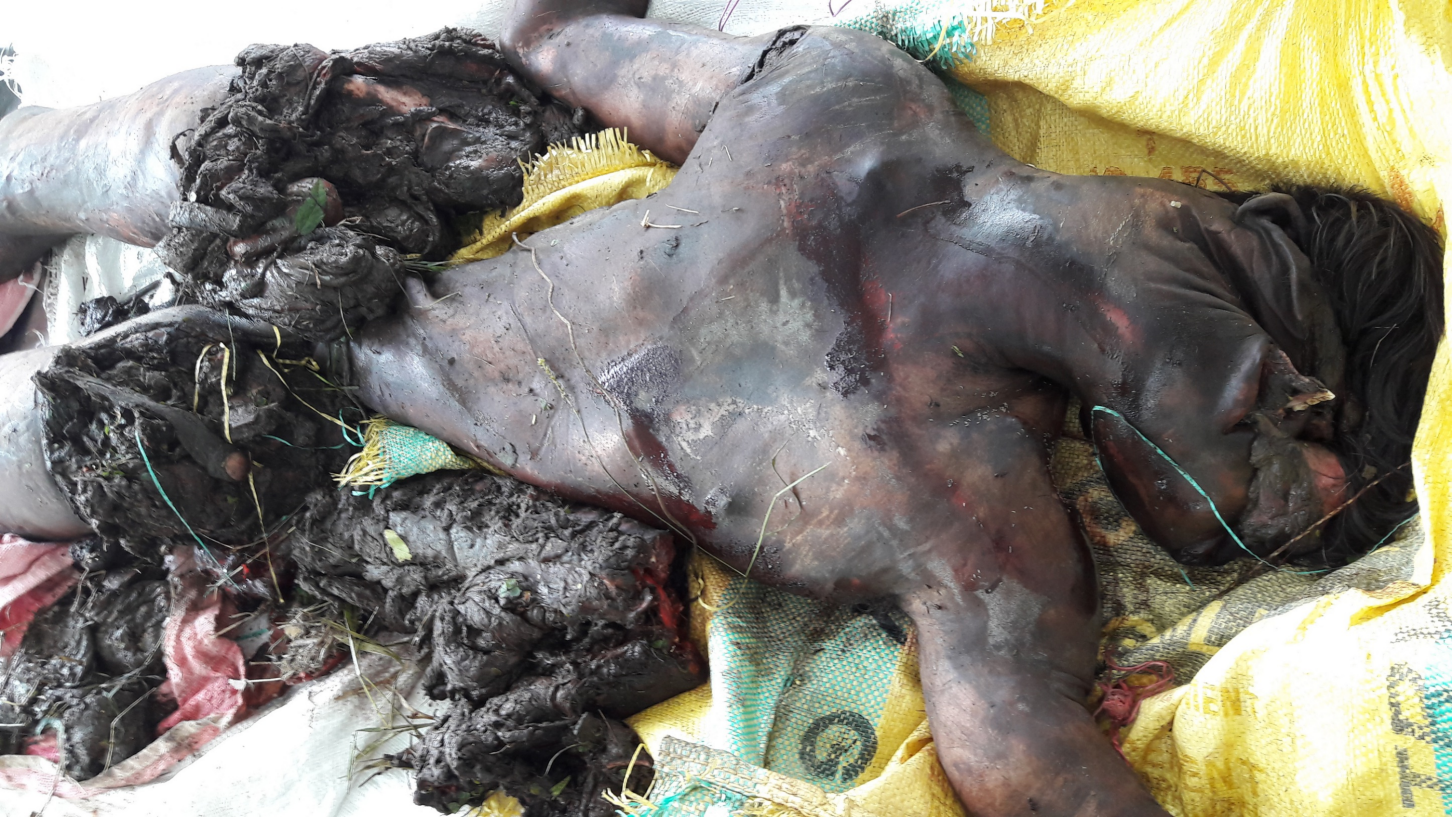
Male victim showing sustained injuries in HEC HEC, human-elephant conflict.

In the majority of fatalities, 21 (91.3%), a head injury and hemorrhagic shock coexisted. A smaller proportion of two (8.7%) had injury only to the head region (Table 4; Figure 4).

**Table 4:** Cause of death in HEC HEC, human-elephant conflict.

Cause of death	Frequency	Percent
Head injury + Hemorrhagic shock	21	91.30
Head injury	2	8.70
Total	23	100.00

Crosstabulation of year of incidence and month of incidence

Interestingly, most fatalities in February, three (75.0%), occurred in 2023, while in December, a significant proportion, one (33.3%), occurred in 2020, a finding that may challenge some preconceived notions. In April, most fatalities, three (60.0%), occurred in 2022. May had the fewest fatalities overall, with one case (20.0%) in 2022. Fatalities in July were distributed equally across 2021, one (20.0%), and 2022, one (9.1%). In September, all fatalities, two (66.7%), occurred in 2020. October recorded the highest number of fatalities overall, with five (45.5%) cases occurring in 2021. In 2020, fatalities were concentrated in September, two (66.7%), and December, one (33.3%). In 2021, fatalities were spread across multiple months, with October having the highest value of five (45.5%). In 2022, fatalities peaked in April, three (60.0%), with additional cases in May and July, one (20.0%) each. In 2023, fatalities were predominantly in February, three (75.0%) (Table 5).

**Table 5:** A crosstabulation by year and month of incidence Note: Values in parentheses indicate the percentage within the respective year of incidence.

Year of Incidence	February	April	May	July	August	September	October	December
2020	0 (0.0%)	0 (0.0%)	0 (0.0%)	0 (0.0%)	0 (0.0%)	2 (66.7%)	0 (0.0%)	1 (33.3%)
2021	1 (9.1%)	1 (9.1%)	0 (0.0%)	1 (9.1%)	0 (0.0%)	0 (0.0%)	5 (45.5%)	3 (27.3%)
2022	0 (0.0%)	3 (60.0%)	1 (20.0%)	1 (20.0%)	1 (20.0%)	0 (0.0%)	0 (0.0%)	0 (0.0%)
2023	3 (75.0%)	0 (0.0%)	0 (0.0%)	0 (0.0%)	0 (0.0%)	0 (0.0%)	0 (0.0%)	0 (0.0%)
Total	4 (17.4%)	4 (17.4%)	1 (4.3%)	2 (8.7%)	1 (4.3%)	2 (8.7%)	5 (21.7%)	4 (17.4%)

For HEC-related fatalities, the relationship between the year and month of incidence was examined by applying a chi-square test of independence. The Pearson chi-square test was statistically significant, χ^2^ (21, N = 23) = 47.44, p = 0.001, suggesting a substantial association with year and month of incidence of double rocking angles. This also shows that the distribution of fatalities is not random and may be determined by the year. Statistical significance was also found in the likelihood ratio chi-square test, χ^2^ (21, N = 23) = 41.43, p = 0.005. Additionally, a significant linear-by-linear association was present, χ^2^ (1, N = 23) = 9.81, p = 0.002, classical test statistic, indicating the possibility of a trend in the association (Table 6).

**Table 6:** Chi-square tests for year of incidence and month of incidence Note: Thirty-two cells (100.0%) have an expected count less than 5. The minimum expected count is 0.13.

Test	Value	df	Asymptotic significance (two-sided)
Pearson chi-square	47.438	21	0.001
Likelihood ratio	41.434	21	0.005
Linear-by-linear association	9.805	1	0.002
N of valid cases	23		

## Discussion

From the 23 victims, the study showed that 52.2% were male and 47.8% were female. The data suggest that among the victims, males were slightly more in number than females. A survey of human-wildlife conflict in Nepal found that men were more likely to be involved in fatal encounters with elephants and leopards than women [7]. The reason is that men's key roles in agricultural and forest activities expose them to wildlife in conflict areas. On the other hand, women are more vulnerable when active at homesteads, such as in water or firewood collection [8].

The majority of victims (26.1%) were between 41 and 50 years old. Other proportional age groups included 31-40 (17.4%) and 51-60 (17.4%). Individuals in the 21-30-year age range (13.0%), 61-70 (13.0%), and over 70 (8.7%) showed smaller percentages. Of the 23 patients, only one was in the 0-10 years age range (4.3%). The HEC fatalities affected the middle-aged, those aged 41-50 years, indicating that it was the most affected age group. HEC incidents disproportionality impact people participating in the agricultural zones in North Bengal, where habitat encroachment and fragmentation are catalytic underlying drivers of conflict. The first group in these activities are middle-aged individuals who expose themselves more to the elephant encounter [9]. Likewise, in Kenya’s Maasai Mara, farming and pastoral communities within and near the protected areas experience increased risk, especially during crop harvests or drought seasons that drive elephants into human-occupied areas, where managing these seasonal risks may require adaptive strategies [10].

The highest proportion of fatalities (47.8%) of all cases occurred in 2021, whereas 21.7% of deaths occurred in 2022, 17.4% in 2023, and 13.0% in 2020. Likewise, in line with the global trend of HEC volatility over the years due to environmental conditions, crop accessibility, and socio-political factors, 2021 saw the highest proportion of HEC deaths. During particular years, crop raiding incidents escalated in Kenya to cause more HEC. Changes in agricultural patterns and elephant behaviors in response to drought or habitat fragmentation were linked to this [11]. With the rate of habitat loss and agricultural intensification being one of the strongest drivers of outbreaks in this species, similar patterns are reported in regions such as Assam, India, where spiking HEC is observed annually [12]. These findings underscore the importance of the efforts at wildlife conservation.

The analysis of HEC fatalities by month displayed marked variation, and October (21.7%) had the most fatalities. Other studies have also shown similar temporal patterns. However, the timing of high conflict is not the same everywhere because of agricultural cycles, elephant migration patterns, and local ecological conditions. Peak conflicts were reported in Assam, India, in the months of rice harvesting (from November to January) and the planting season (April to June) [13]. The region where these conflicts occur is North Bengal, India, an area with high HEC, which is characterized by increasing conflicts in the post-monsoon period and is associated with elephant migration and human activities near forest fringes. Every time of year, this pattern matches seasonal changes in resource availability (food and water), which is reassuringly matched with our research [9]. However, these studies do not explicitly address the possibility that some patterns, such as our observation of October fatalities, may arise from regional characteristics, e.g., local harvest times or changes in forest cover, leading elephants to congregate closer to human settlements at this time of year.

According to the current study, most HEC fatalities (91.3%) took place at the site of the incident due to the sudden and often violent nature of elephant attacks. Most of the HEC fatalities in Assam happened immediately at the site because of severe and usually fatal trampling or tusk strikes. The time of these events is rapid, and the location of many is remote, resulting in limited access to emergency medical care [14]. In North Bengal, most fatalities were instantaneous - either during nighttime raids on crops or settlements or by repeated stabbings and hackings. Because of the severity of the injuries (and the rough terrain), victims seldom made it to medical facilities [9].

HEC fatalities (47.8%) occurred during the late afternoon and evening hours (4 PM to 12 AM). This aligns with other studies' findings concerning temporal patterns of human-wildlife interactions. During these times, elephants raid crops and, in turn, factions. In Kenya, as elsewhere, wildlife was found to be most active during periods of the day when activity was at its greatest - like the early evening. Both of these periods overlap when humans return home or guard fields, which increases the potential for conflict [15]. Similar to Sri Lanka, research shows that many HEC incidents occurred in the latter portions of the evening. Elephants moving closer to human settlements at night because of the cover of darkness to attain food was given as the reason [16].

The research shows that the major cause of death (91.3%) in the HEC fatalities was head injury and hemorrhagic shock, consistent with the patterns seen in other studies. As elephants are big, powerful animals, human victims of these encounters almost always suffer severe blunt-force trauma in the form of being struck or trampled. A study of HECs in Assam found that the primary cause of death was head injuries from elephants’ tusks striking or trampling the caretaker. The typical outcome in these incidents is a combination of traumatic injuries and hemorrhagic shock secondary to the sheer force of such attacks [13]. In Sri Lanka, many fatalities occurred from crushing or injuries to the head and torso from tusk injuries. Moreover, victims are often severely bleeding (hemorrhagic shock), and they are usually not able to be seen immediately in remote areas [16]. HEC was found to be in the form of head injuries and internal bleeding, most frequently after elephant attacks in agricultural and pastoral regions of Kenya, where it is prevalent. These injuries are often severe, and so routinely result in rapid death, with few surviving long enough to get to medical facilities [15].

Local tribes in this region of Jharkhand keep rice beer, mahua wine, and other homemade alcoholic drinks. These drinks are prepared after storing rice and wheat long, which attracts elephants and leads to severe incidents. The forest department is making villagers in the Hazaribagh area, which is prone to elephant attacks, aware of the elephant app. This app will help reduce the loss of life and property [17].

However, the Forest Department has advised several safety rules for protection against wild elephants. These guidelines include immediate notification of elephant entry into the area to the forest management department and recommendations for behavior in hilly terrain or when meeting an elephant. One might throw clothes and run in a zigzag pattern to distract an elephant. Adding red chili powder to burned motor oil or grease and tying it in a thick rope can be a barrier by separating people from elephants. The second method is burning red chili powder and dried cow dung cakes. Before an elephant attack, it flings up both the ear and trunk. A communication network and awareness campaigns with the local communities, forest dwellers, and the forest department are vital. However, it is important to emphasize that a diverse and practical forest cover provides safety to elephants and is essential to their conservation. To be effective, the mitigation of conflict must address the root causes of conflict. The effective management of corridors can make an enormous positive difference to people and elephants. Thus, the present findings will aid governmental and nongovernmental organizations in designing and implementing corral management, conservation actions, and the welfare of tribal communities [18].

Limitations

There might be bias in the data collection process through its limited four years of data collection and in just one district. Autopsies were performed at the tertiary center's study site on the fatalities brought to the tertiary center. Additionally, a patient's referral to a higher-level center in the state may have affected the observed result. Such bias requires urgent addressing in future studies and will need more comprehensive and inclusive design.

## Conclusions

Human and elephant populations are at risk due to their involvement in conflicts. Human activity caused these conflicts to become more frequent, and their environment and resources were significantly altered. As outlined in this article, sheaths are utilized to overcome these issues. Humans must take action to prevent such conflicts as the first and most significant step.

Addressing the root causes of such conflict is an essential first step toward conflict mitigation. Establishing a good communication network and awareness programs involving local people, forest dwellers, and forest departments is one of the major safety measures. The emphasis on developing a more variable and feasible dense forest cover is essential for the elephants. Installing beehives and chilly fences proved to be very useful to prevent conflicts. Effective management of corridors would go a long way in ensuring a symbiotic relationship between elephants and human beings. Therefore, the present findings will be helpful for the government and non-government bodies for the improvement of the corridors, their conservation, management, and livelihood of the forest-dependent population.
